# Deep learning for quality assessment of optical coherence tomography angiography images

**DOI:** 10.1038/s41598-022-17709-8

**Published:** 2022-08-12

**Authors:** Rahul M. Dhodapkar, Emily Li, Kristen Nwanyanwu, Ron Adelman, Smita Krishnaswamy, Jay C. Wang

**Affiliations:** 1 Department of Ophthalmology, Yale School of Medicine, New Haven, CT 06510 USA; 2 Division of Oculoplastics and Reconstructive Surgery, Wilmer Eye Institute, Baltimore, MD 21287 USA; 3 Department of Genetics, Yale School of Medicine, New Haven, CT 06510 USA; 4 Department of Computer Science, Yale University, New Haven, CT 06510 USA; 5 Northern California Retina Vitreous Associates, Mountain View, CA 94040 USA

**Keywords:** Translational research, Medical imaging

## Abstract

Optical coherence tomography angiography (OCTA) is an emerging non-invasive technique for imaging the retinal vasculature. While there are many promising clinical applications for OCTA, determination of image quality remains a challenge. We developed a deep learning-based system using a ResNet152 neural network classifier, pretrained using ImageNet, to classify images of the superficial capillary plexus in 347 scans from 134 patients. Images were also manually graded by two independent graders as a ground truth for the supervised learning models. Because requirements for image quality may vary depending on the clinical or research setting, two models were trained—one to identify high-quality images and one to identify low-quality images. Our neural network models demonstrated outstanding area under the curve (AUC) metrics for both low quality image identification (AUC = 0.99, 95%CI 0.98–1.00, $\kappa$ = 0.90) and high quality image identification (AUC = 0.97, 95%CI 0.96–0.99, $\kappa$ = 0.81), significantly outperforming machine-reported signal strength (AUC = 0.82, 95%CI 0.77–0.86, $\kappa$= 0.52 and AUC = 0.78, 95%CI 0.73–0.83, $\kappa$ = 0.27 respectively). Our study demonstrates that techniques from machine learning may be used to develop flexible and robust methods for quality control of OCTA images.

## Introduction

Optical coherence tomography angiography (OCTA) is a relatively new technology based on optical coherence tomography (OCT) that may be used to noninvasively visualize the retinal microvasculature. OCTA measures the difference in reflectance patterns from repeated pulses of light to the same region of the retina, which can then be computationally reconstructed to show blood vessels without the invasive use of dyes or other contrast agents. OCTA also allows for depth-resolved vessel imaging, enabling clinicians to separately inspect the superficial and deep vascular layers, which can be useful for differentiating between chorioretinal diseases.

While this technology holds much promise, variation in image quality remains a major problem for reliable image analysis, making interpretation of images more challenging and hindering widespread clinical adoption. As OCTA relies on multiple sequential OCT scans, it is more susceptible to image artifacts than standard OCT. Most commercially available OCTA platforms provide a proprietary measure of image quality termed signal strength (SS) or sometimes signal strength index (SSI). An image with a high SS or SSI, however, does not guarantee the absence of image artifacts, which can compromise any subsequent image analysis and may lead to inappropriate clinical decisions. Common image artifacts that can occur in acquisition of OCTA images include motion artifacts, segmentation artifacts, media opacity related artifacts, and projection artifacts^1–3^.

As OCTA-derived metrics such as vessel density are being increasingly used for translational research, clinical trials, and in clinical practice, there is a great need to develop reliable and robust processes for image quality control that can account for the presence of image artifacts^4^. Skip connections, also known as residual connections, are projections in a neural network architecture that allow information to bypass convolutional layers, preserving information at multiple scales or resolutions^5^. Because image artifacts may affect both small-scale and overall large-scale features of an image, a neural network with skip connections is ideally suited for automation of this quality control task^5^. Recently published work has shown some promise using deep convolutional neural networks trained with quality data generated by human graders^6^.

In this study, we train convolutional neural networks with skip connections to perform automated quality determination for OCTA images. We build upon previous work by developing separate models for identifying high-quality images and low-quality images because requirements for image quality may vary depending on the particular clinical or research scenario. We compare the results of these networks to convolutional neural networks without skip connections to assess the value of incorporating features at multiple levels of granularity in a deep learning framework. We then benchmark our results against signal strength, a commonly used manufacturer-provided measurement of image quality.

## Methods

### Patient enrollment

Patients with diabetes seen at the Yale Eye Center between 8/11/2017 and 4/11/2019 were included in our study. Patients with any non-diabetic chorioretinal disease were excluded. There were no inclusion or exclusion criteria on the basis of age, sex, ethnicity, image quality, or any other factor.

### Image collection

OCTA images were obtained using the AngioPlex platform on the Cirrus HD-OCT 5000 (Carl Zeiss Meditec Inc, Dublin, CA) within the 8 $\times$ 8 mm and 6 $\times$ 6 mm imaging protocols. Informed consent was obtained by each study participant for participation in the study and The Institutional Review Board (IRB) at Yale approved the use of a global photography informed consent for all patient data. The tenets of the Declaration of Helsinki were followed. The study was approved by the IRB at Yale.

### Image analysis

Superficial slab images were graded based on a previously described motion artifact score (MAS), a previously described segmentation artifact score (SAS), centration on the fovea, presence of media opacity, and good visualization of small capillaries as determined by the image grader^7^. Images were analyzed by two independent graders (R.D. and J.W.). Images were assigned a gradability score of 2 (gradable) if all the following criteria were satisfied: image was centered on the fovea (less than 100 pixels from the image center), MAS was 1 or 2, SAS was 1, a media opacity was present in less than 1/16 of the image, and small capillaries were visible in greater than 15/16 of the image. Images were assigned a gradability score of 0 (not gradable) if any of the following criteria were satisfied: the image was decentered, if the MAS was 4, if the SAS was 2, or if a media opacity was present in greater than 1/4 of the image and small capillaries could not be distinguished in greater than 1/4 of the image. All other images that did not meet criteria for either a gradability score of 0 or 2 were assigned a gradability score of 1 (borderline).

**Figure 1:**
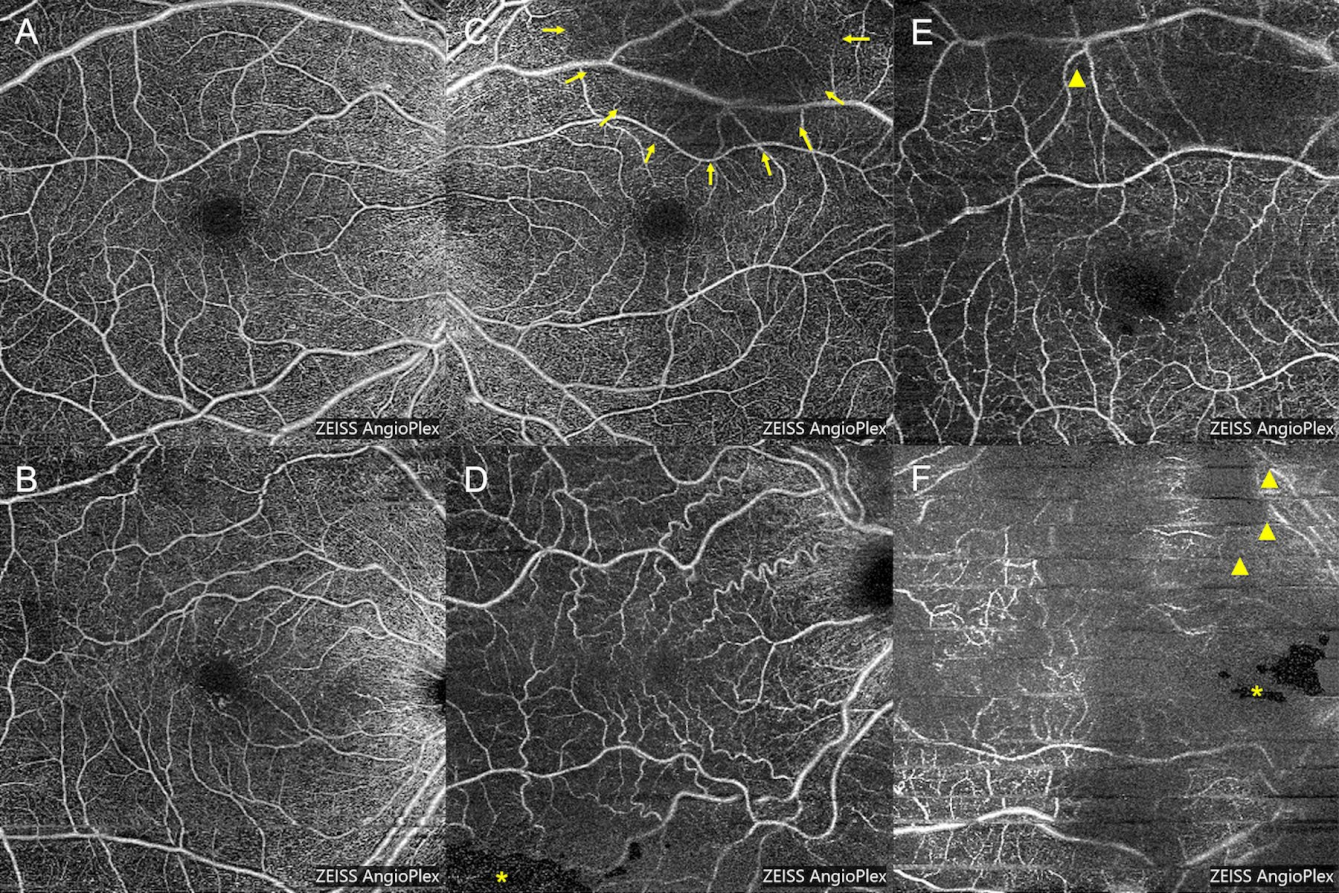
Representative examples of 8 × 8 mm OCTA images of the superficial capillary plexus with gradability score of 2 (**A,B**), 1 (**C,D**), and 0 (**E,F**). Image artifacts displayed include blink lines (arrowheads), segmentation artifact (asterisks), and media opacity (arrows). Image (**E**) is also decentered.

Figure 1 shows examples of images of each gradability score and image artifacts. Inter-rater reliability of individual gradings was assessed by weighted Cohen’s kappa^8^. Individual scores from each grader were summed to create a total gradability score for each image ranging from 0 to 4. Images with a combined score of 4 were considered high quality. Images with a combined score of 0 or 1 were considered low quality.

### Deep learning framework

A convolutional neural network with the ResNet152 architecture (Fig. 3A.i) pre-trained on images from the ImageNet database was generated using the fast.ai and PyTorch frameworks^5,9–11^. A convolutional neural network is one which uses learned filters that scan through image patches to learn spatially localized features. Our trained ResNet is a neural network with 152 layers, which prominently feature skip or “residual connections”, which simultaneously carry information at many resolutions. By projecting information through the network from multiple resolutions, the framework may learn features of low quality images at multiple levels of granularity. Alongside our ResNet model, we also trained AlexNet, a well-studied neural network architecture without skip connections as a comparison (Fig. 3A.ii)^12^. Without skip connections, this network would be limited in its ability to capture features at finer granularities.

The original set of 8 $\times$ 8 mm OCTA images was augmented using both horizontal and vertical flip techniques^13^. The full dataset was then split randomly at the image level into train (51.2%), test (12.8%), hyperparameter tuning (16%) and validation (20%) datasets using the scikit-learn python toolkit^14^. Two scenarios were considered - one based on identifying only the highest quality images (total gradability score of 4) and another based on identifying only the lowest quality images (total gradability score of 0 or 1). The neural network was retrained on our image data once for each of the high quality and low quality use cases. In each use case, the neural network was trained for 10 epochs with all but the most high order layer weights frozen, and for 40 epochs with learning proceeding on all internal parameter weights using a discriminative learning rate approach with a cross-entropy loss function^10,15,16^. A cross-entropy loss function is a log-scaled measurement of the mismatch between network predicted labels and ground truth. During training, gradient descent was performed on the internal parameters of the neural network to minimize loss. Learning rate, dropout rate, and weight decay hyperparameters were tuned using Bayesian optimization with 2 random starting points and 10 iterations, using AUC on the hyperparameter tuning dataset as an objective^17^.

Receiver operating characteristic (ROC) curves were then generated for all neural network models as well as machine-reported signal strength for each of the low quality and high quality use cases. Area under the curve (AUC) was calculated using the pROC R package and 95% confidence intervals and p-values were calculated using DeLong’s method^18,19^. The total human-rater gradability score was used as ground truth for all ROC calculations. For machine reported signal strength, AUC was calculated twice: once against the high-quality gradability score cutoff and once against the low-quality gradability score cutoff. Neural networks were compared to a signal strength AUC mirroring their own training and evaluation conditions.

For additional validation of the trained deep learning models on a separate data set, both high quality and low quality models were directly applied to assess quality of 32 en-face 6 $\times$ 6 mm superficial slab images gathered from the Yale Eye Center during the same period as the 8 $\times$ 8 mm images. These 6 $\times$ 6 mm images were manually graded by the same raters (R.D. and J.W.) in the same manner as the 8 $\times$ 8 mm images, and AUC as well as accuracy and Cohen’s kappa were similarly calculated.

Class imbalance ratios were 158:189 ($\rho = 1.19$) for the low quality model and 80:267 ($\rho = 3.3$) for the high quality model. As the class imbalance ratio did not exceed 1:4, no specific architectural changes were made to correct for class imbalance^20,21^.

To better visualize the learning process, class activation maps were generated for all four deep learning models trained: the high quality ResNet152 model, the low quality ResNet152 model, the high quality AlexNet model, and the low quality AlexNet model. The class activation maps were generated from the input convolutional layers of these four models and heatmaps were produced by overlaying the activation maps with their source images from the 8 × 8 mm and 6 × 6 mm validation sets^22,23^.

R version 4.0.3 was used for all statistical calculations and visualizations were generated using the ggplot2 library of graphical tools^24^.

## Results

### Image characteristics

We collected 347 en-face 8 $\times$ 8 mm images of the superficial capillary plexus from 134 subjects. Machine-reported signal strength between 0 and 10 was available for all images (mean = 6.99 ± 2.29). Of the 347 images obtained, the average age at time of exam was 58.7 ± 14.6 years old and 39.2% were from male patients. Of all images, 30.8% were from Caucasian patients, 32.6% from Black patients, 30.8% from Hispanic patients, 4% from Asian patients and 1.7% from patients of other ethnicities (Table 1). The distribution of patient age at time of OCTA varied significantly with image quality (p < 0.001). The percentage of high quality images from younger patients aged 18–45 was 33.8% as compared to 12.2% of low quality images (Table 1). The distribution of diabetic retinopathy status also varied significantly with image quality (p < 0.017). Of all high quality images, the percentage from patients with PDR was 18.8% as compared to 38.8% of all low quality images (Table 1).

**Figure 2:**
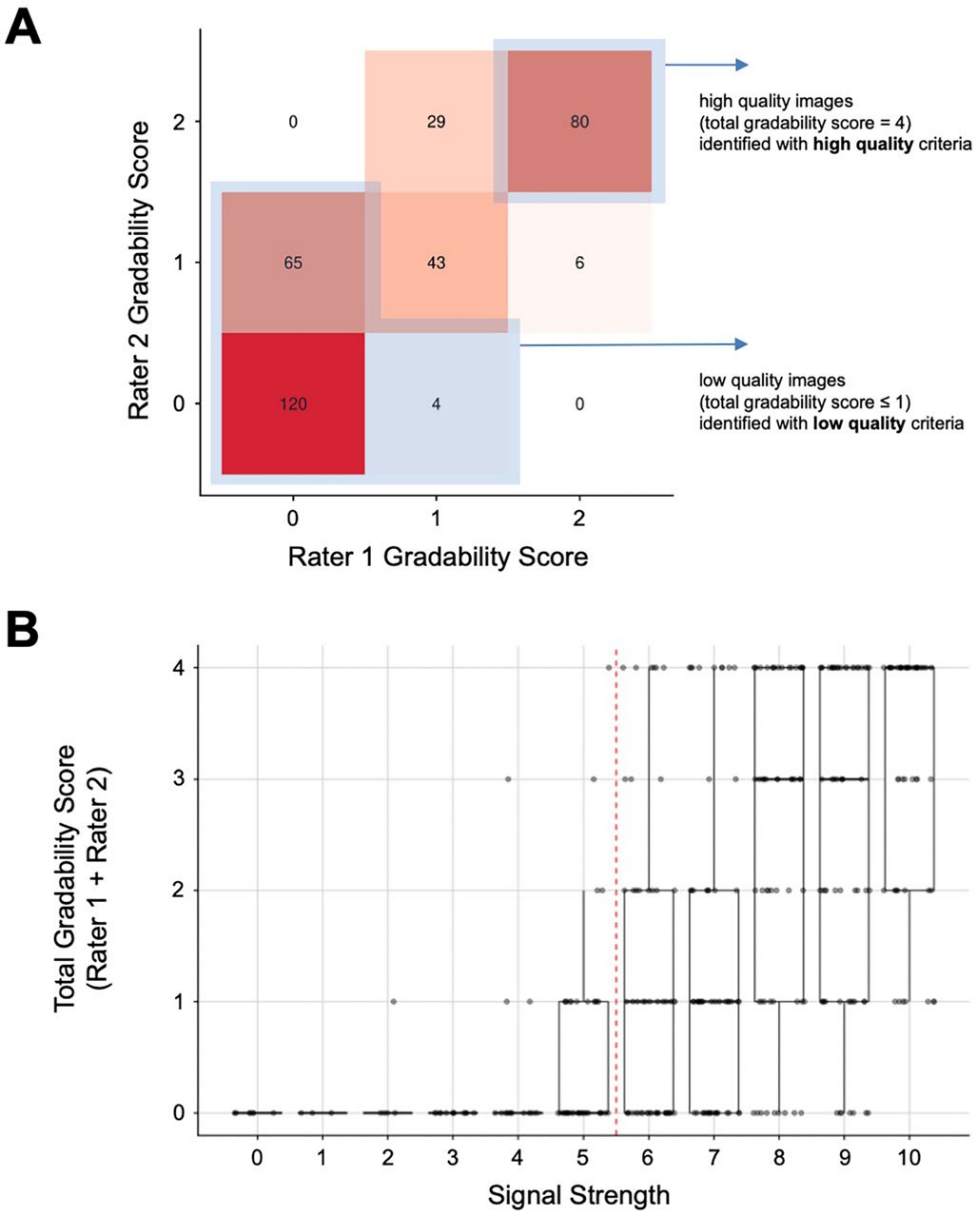
Plot showing gradability scores for each independent grader and comparison with machine-reported signal strength. (**A**) The sum of gradability scores was used to create a total gradability score. Images with total gradability score of 4 were designated as high quality, and those with total gradability score of less than or equal to 1 were designated as low quality. (**B**) Signal strength is correlated with manual gradability score, but images of high signal strength may still be of low quality. Red dashed line indicates manufacturer recommended signal strength based quality cutoff (signal strength $\ge$ 6).

Individual gradability ratings of all images showed a moderate to strong degree of inter-rater reliability between human image readers (weighted Cohen’s kappa = 0.79, 95% CI: 0.76-0.82), and there were no images in which graders differed by more than one point (Fig. 2A). Signal strength was significantly correlated with manual gradability score (Pearson’s product-moment correlation = 0.58, 95% CI 0.51–0.65, p < 0.001) but numerous images were identified with high signal strength but low manual gradability score (Fig. 2B).

**Table 1:** Patient characteristics for OCTA images show that patient age and diabetic retinopathy status is significantly associated with image quality, but sex and ethnicity are not.

	All images	Low quality images (total gradability score $\le$ 1)	High quality images (total gradability score $=$ 4)	p-value
**Total**	347	189	80	
**Age**				< 0.001
18-44	68 (19.6%)	23 (12.2 %)	27 (33.8%)	
45-64	165 (47.6%)	92 (48.7%)	35 (43.8%)	
65+	114 (32.9%)	74 (39.2%)	18 (22.5%)	
**Sex**				0.52
Male	136 (39.2%)	71 (37.6%)	36 (45.0%)	
Female	211 (60.8%)	118 (62.4%)	44 (55.0%)	
**Ethnicity**				0.37
Caucasian	107 (30.8%)	59 (31.2%)	29 (36.3%)	
Black	113 (32.6%)	59 (31.2%)	19 (23.8%)	
Hispanic	107 (30.8%)	64 (33.9%)	23 (28.8%)	
Asian	14 (4.0%)	5 (2.6%)	7 (8.8%)	
Other	6 (1.7%)	2 (1.1%)	2 (2.5%)	
DR Status*				0.017
None	105 (30.8%)	50 (27.3%)	27 (33.8%)	
NPDR	139 (40.8%)	62 (33.9%)	38 (47.5%)	
PDR	97 (28.4%)	71 (38.8%)	15 (18.8%)	

P-values were calculated using the chi-square statistic.

*DR* diabetic retinopathy,* NPDR* nonproliferative diabetic retinopathy,* PDR* proliferative diabetic retinopathy.

* Diabetic retinopathy status could not be accurately determined for 6 images, which have been omitted.

### Performance of the deep learning framework

**Figure 3:**
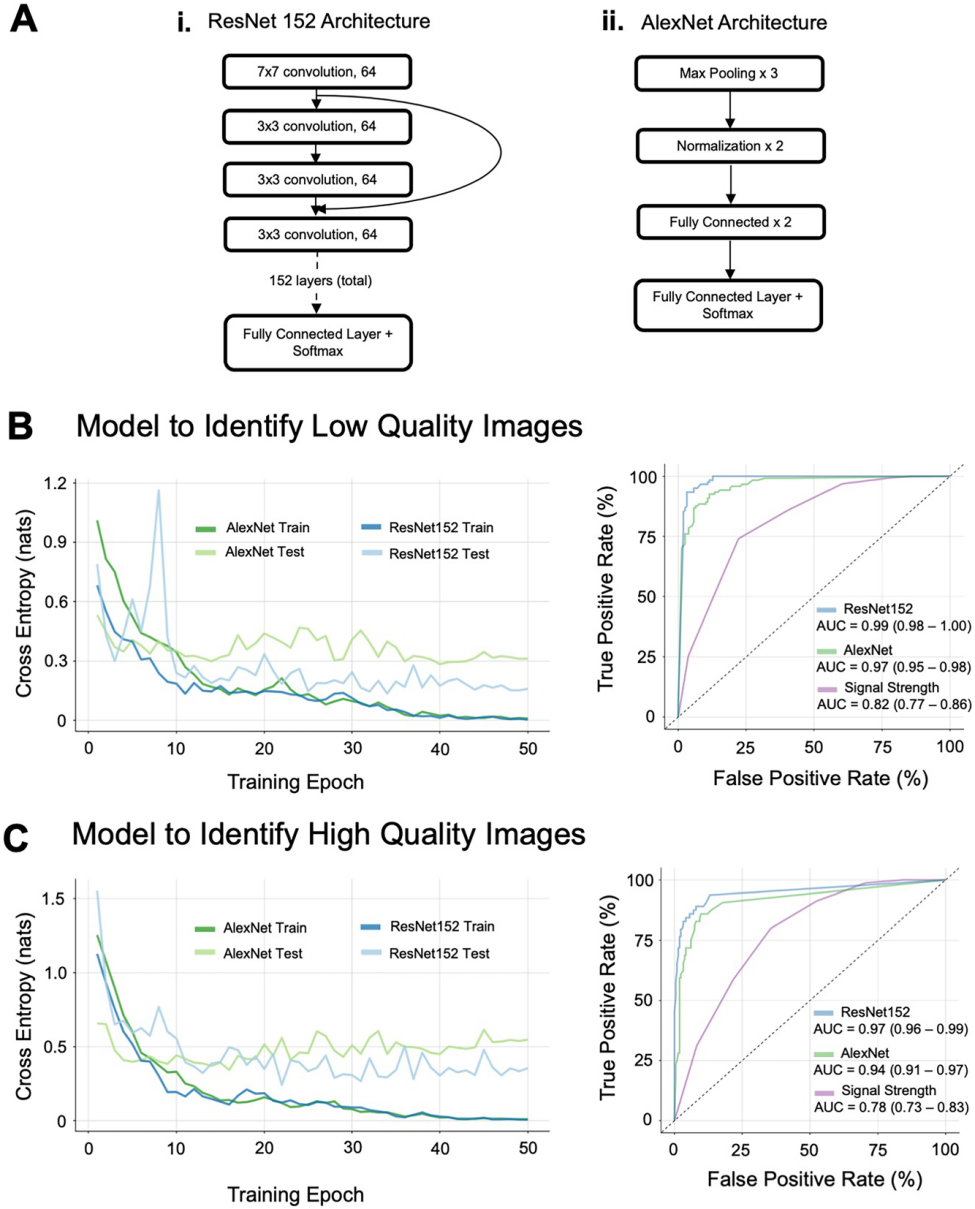
ResNet transfer learning achieves significant improvements in identification of image quality in both low quality and high quality use cases over machine-reported signal strength. (**A**) Simplified architecture diagrams for the pretrained (i) ResNet152 and (ii) AlexNet architectures employed. (**B**) Training history and receiver operating characteristic curves for ResNet152 versus machine-reported signal strength and AlexNet for low quality criteria. (**C**) Training history and receiver operating characteristic curves for ResNet152 versus machine-reported signal strength and AlexNet for high quality criteria.

During training of both the ResNet152 and AlexNet architectures, validation and training cross-entropy losses declined through a 50 epoch training sequence (Fig. 3B,C). Validation accuracy at the final training epoch exceeded 90% for both the high quality and the low quality use cases.

Receiver operating characteristic curves showed that the ResNet152 model significantly outperformed machine-reported signal strength (p < 0.001) in both the low quality and high quality use cases. The ResNet152 model also significantly outperformed the AlexNet architecture (p = 0.005 and p = 0.014 for the low quality and high quality cases respectively). The models generated for each of these tasks were able to obtain AUC values of 0.99 and 0.97 respectively, significantly superior to respective AUC values of 0.82 and 0.78 for machine-reported signal strength index or 0.97 and 0.94 for AlexNet (Fig. 3). The difference between the ResNet and signal strength AUCs was higher in the identification of high quality images, indicating that there was more added benefit to using ResNet for this task.

Maximal prediction accuracy for the ResNet152 model after tuning decision boundary threshold was 95.3% for the low quality use case and 93.5% for the high quality use case (Table 2). The maximal prediction accuracy for the AlexNet model was 91.0% for the low quality use case and 90.1% for the high quality use case (Table 2). The maximal prediction accuracy for signal strength was 76.1% for the low quality use case and 77.8% for the high quality use case. Agreement between the ResNet152 models and human graders as measured by Cohen’s kappa ($\kappa$) was 0.90 for the low quality use case and 0.81 for the high quality use case. Cohen’s kappa for AlexNet was 0.82 and 0.71 for low quality and high quality use cases respectively. Cohen’s kappa for signal strength was 0.52 and 0.27 for low quality and high quality use cases respectively.

Validation of the high quality identification and low quality identification models against 6 $\times$ 6 mm superficial slab images demonstrated the ability of the trained models to determine image quality across different image acquisition parameters. When used to assess quality of the 6 $\times$ 6 mm superficial slab images, the low quality model achieved an AUC of 0.83 (95% CI: 0.69-0.98), while the high quality model achieved an AUC of 0.85 (95% CI: 0.55-1.00) (Table 2).

**Table 2:** Validation of low quality and high quality ResNet models trained on 8 $\times$ 8 mm images against 8 $\times$ 8 mm and 6 $\times$ 6 mm OCTA superficial slab images shows robust quality assessment.

	8 $\times$ 8 mm validation set (n = 69)	6 $\times$ 6 mm validation set (n = 32)
Low quality ResNet (trained on low quality 8 $\times$ 8 mm image cutoff)	AUC = 0.99, 95% CI [0.98–1.00] Accuracy = 95.3% Cohen’s Kappa = 0.90	AUC = 0.83, 95% CI [0.69–0.98] Accuracy = 87.5% Cohen’s Kappa = 0.7
Low quality CNN (trained on low quality 8 $\times$ 8 mm image cutoff)	AUC = 0.97, 95% CI [0.95–0.98] Accuracy = 91 % Cohen’s Kappa = 0.82	AUC = 0.79, 95% CI [0.63–0.96] Accuracy = 68.8% Cohen’s Kappa = 38.2
High quality ResNet (trained on high quality 8 $\times$ 8 mm image cutoff)	AUC = 0.97, 95% CI [0.96–0.99] Accuracy = 93.5% Cohen’s Kappa = 0.81	AUC = 0.85, 95% CI [0.55–1.00] Accuracy = 71.9% Cohen’s Kappa = 0.41
High quality CNN (trained on high quality 8 $\times$ 8 mm image cutoff)	AUC = 0.94, 95% CI [0.91–0.97] Accuracy = 90.1% Cohen’s Kappa = 0.71	AUC = 0.67, 95% CI [0.19–1.00] Accuracy = 71.8% Cohen’s Kappa = 0.41

*AUC* area under the curve.

**Figure 4:**
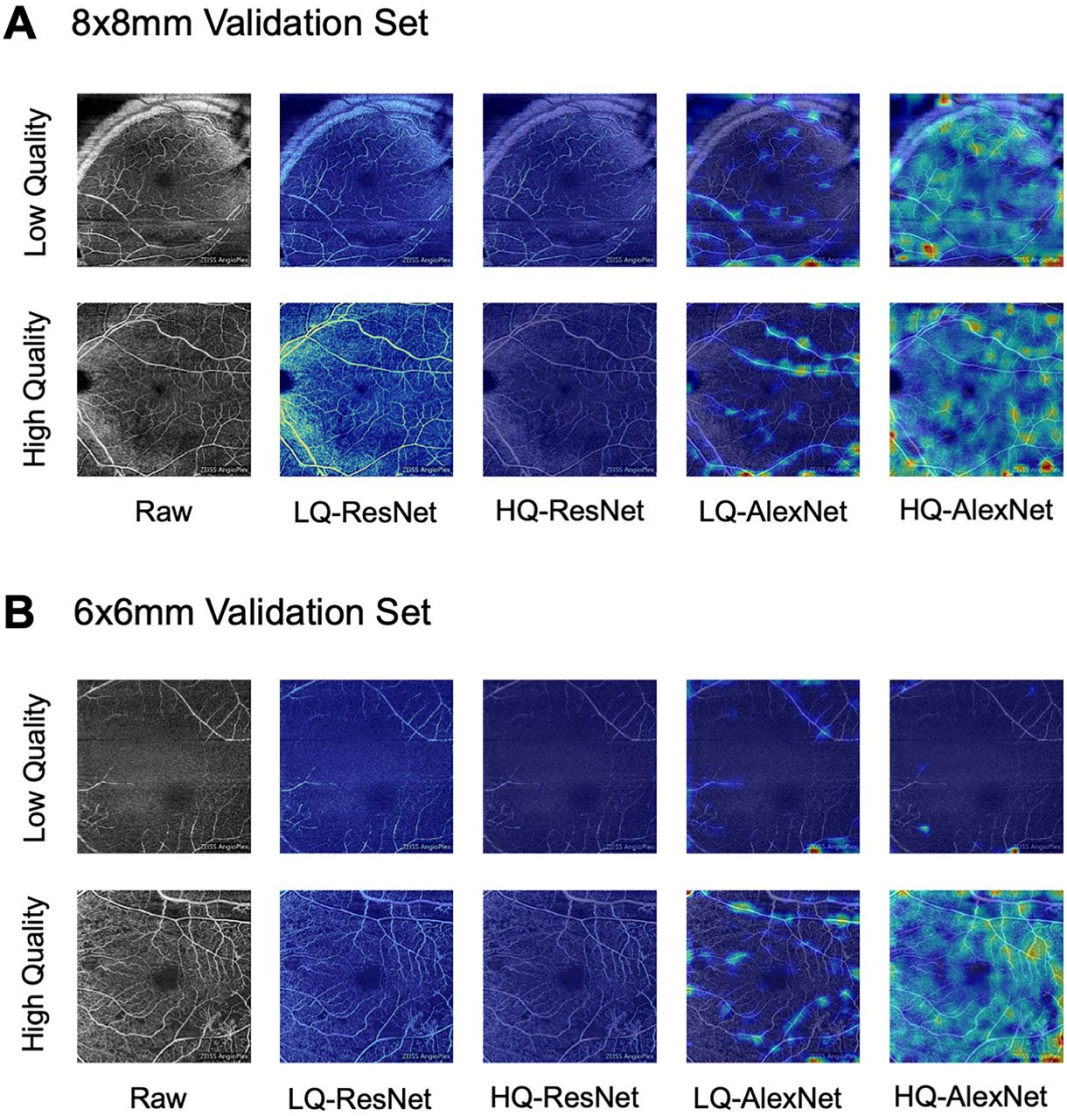
Class activation maps for ResNet152 and AlexNet models highlight features associated with image quality. (**A**) Class activation maps show coherent activation following the superficial retinal vasculature in the 8 $\times$ 8 mm validation images and (**B**) to a lesser degree in 6 $\times$ 6 mm validation images.* LQ* model trained with low-quality criteria,* HQ* model trained with high-quality criteria.

Visual inspection of the input layer class activation maps show that all trained neural networks use salient image features during image classification (Fig 4A,B). For both the 8 $\times$ 8 mm and 6 $\times$ 6 mm images, the ResNet activation maps closely follow the retinal vascular structure. The AlexNet activation maps also follow the retinal vessels but at coarser resolution.

## Discussion

It has been previously shown that image quality can greatly affect any quantitative measurements of OCTA images^25^. In addition, the presence of retinal pathology can increase the prevalence of image artifacts^7,26^. Indeed, in our data, consistent with previous studies, we found a significant correlation between increasing age and severity of retinal disease with poorer image quality (p < 0.001, p = 0.017 for age and DR status respectively; Table 1)^27^. Thus, prior to any quantitative analysis of OCTA imaging, assessment of image quality is paramount. Most studies analyzing OCTA images use a threshold of machine-reported signal strength to exclude images of poor quality. While signal strength has been shown to affect quantification of OCTA parameters, high signal strength alone may not be sufficient to exclude images with image artifacts^2,3,28,29^. Thus, there is a need to develop a more robust method of image quality control. To that end, we assessed the performance of a supervised deep learning approach compared to machine-reported signal strength.

We developed more than one model for assessing image quality because different scenarios in which OCTA is used may have varying requirements for image quality. For instance, images must be of higher quality if the output of interest is continuous (e.g. vessel density or fractal dimension in a research or clinical trial setting) versus binary (e.g. presence of choroidal or preretinal neovascularization in a clinical setting). Furthermore, the particular quantitative parameter of interest is also of consequence. For example, foveal avascular zone area will not be affected by a non-central media opacity but vessel density would be impacted. While our study remains aimed at a general approach to image quality—not tied to the requirements of any one particular test but rather designed as a drop-in replacement for machine-reported signal-strength—we hope to provide users a greater degree of control such that they may choose a model matching the maximum degree of image artifact that is considered acceptable, depending on a user’s particular metric of interest.

For both low quality and high quality scenarios, we demonstrate excellent performance of deep convolutional neural networks utilizing skip connections in the quality assessment of 8 $\times$ 8 mm OCTA images of the superficial capillary plexus with AUCs of 0.97 and 0.99 for high and low quality models respectively. We also show superior performance of our deep learning approach as compared to using machine-reported signal strength alone. Skip connections allow neural networks to learn features at multiple levels of granularity, capturing fine-grain aspects of images such as contrast as well as overall features such as image centering^30,31^. Because image artifacts that affect image quality may be best identified across a wide range of scales, neural network architectures with skip connections may exhibit superior performance to those without for the task of image quality determination.

While testing our models on 6 $\times$ 6 mm OCTA images, a different size than the models were trained to classify, we noted a reduction in classification performance for both the high quality and low quality models (Fig. 2). This drop was larger for the AlexNet models as compared to the ResNet models. The relatively better performance of ResNet may be due to the ability of residual connections to carry information at multiple scales, thereby rendering the models more robust to classifying images taken at multiple scales and/or magnifications.

Some differences between the 8 $\times$ 8 mm images and 6 $\times$ 6 mm images that may contribute to decreased classification performance include a relatively larger proportion of the image comprising the foveal avascular zone, changes to the visibility of the vascular arcades, and absence of the optic nerve from the 6x6mm images. Regardless, the ability of our high quality ResNet model to achieve an AUC of 85% for 6 $\times$ 6 mm images, a configuration for which the model was not trained, is suggestive that the image quality information encoded in the neural network is applicable beyond the single image size or machine configuration for which it was trained (Table 2). Reassuringly, the ResNet and AlexNet class activation maps from the 8 $\times$ 8 mm and 6 $\times$ 6 mm images are able to highlight the retinal vessels in both cases, suggesting that the models have learned important information applicable to the classification of both types of OCTA images (Fig. 4).

Lauermann et al. similarly used deep learning approaches for image quality assessment of OCTA images using the Inception architecture, a different convolutional neural network with skip connections^6,32^. They similarly limited their study to images of the superficial capillary plexus but only used smaller images of 3 × 3 mm from the Optovue AngioVue though patients with different chorioretinal diseases were also included. Our work builds on theirs by including multiple models to address different image quality cutoffs and validating the results across multiple image sizes. We additionally report AUC metrics for our machine learning models and improve on their already impressive accuracy (90%) in both our low quality (96%) and high quality (95.7%) models^6^.

There are several limitations to this study. First, images were taken on only one OCTA machine, and only 8 $\times$ 8 mm and 6 $\times$ 6 mm images of the superficial capillary plexus were included. The reason for excluding images from deeper layers is that projection artifact would have made manual grading of images more difficult and potentially less consistent. In addition, images were taken only from diabetic patients, a patient population for whom OCTA is emerging as an important diagnostic and prognostic tool^33,34^. While we were able to validate our models on images of a separate size to ensure robustness of the results, we were unable to identify a suitable dataset from a different center, limiting our assessment of model generalizability. While only from a single center, our images came from patients with diverse ethnic and racial backgrounds, a unique strength of our study. By incorporating diversity into our training process, we hope that our results may be more broadly generalizable, and we may avoid encoding racial bias in our trained models.

Our study demonstrates that neural networks with skip connections can be trained to achieve high performance in the determination of OCTA image quality. We provide these models as a tool for further study. Because different quantitative metrics may have different requirements for image quality, separate quality control models could be developed for each metric using the framework established here.

Future study should include images of various sizes, from different depths, and from different OCTA machines to obtain a deep learning image quality grading process that is generalizable across OCTA platforms and imaging protocols. The current study also relies on supervised approaches to deep learning which require manual assessment and grading of images, which can be laborious and time intensive for large data sets. It remains to be seen whether unsupervised approaches to deep learning can adequately separate images of poor from high quality.

As OCTA technology continues to evolve and the speed of scanning improves, the frequency of imaging artifacts and poor-quality images is likely to decrease. Software advances such as the recent introduction of projection artifact removal will likely mitigate such limitations as well. Nevertheless, numerous challenges remain, as imaging patients with poor fixation or with significant media opacities will invariably result in image artifacts. As OCTA becomes more widely used in clinical trials, careful deliberation is needed to establish clear guidelines for the degree of image artifact that is considered acceptable for image analysis. The application of deep learning approaches to OCTA images holds great promise and further study in this area is needed to develop a robust approach to image quality control.

## Data Availability

The code used during the current study is available in the octa-qc repository, https://github.com/rahuldhodapkar/octa-qc. The datasets generated during and/or analysed during the current study are available from the corresponding author on reasonable request.
